# Art Therapy Experiential Workshops During Work Hours to Promote Doctors’ Wellbeing – A Quantitative Analysis

**DOI:** 10.1192/bjo.2025.10323

**Published:** 2025-06-20

**Authors:** Safia Zaffarullah, Claire O’Donoghue, Richard Whitaker

**Affiliations:** Surrey and Borders Partnership NHS Foundation Trust, Surrey, United Kingdom

## Abstract

**Aims:** Risk of burnout amongst Resident Doctors is higher now than even pandemic levels with 63% of trainees at moderate-high risk in the 2024 GMC NTS. With an ever-increasing need to consider novel approaches to tackle this, Art Therapy (AT) “experiential” workshops were delivered for attendees of the weekly academic programme at SABP NHS Foundation Trust.

The primary aim was to explore whether engaging in AT could positively impact doctors in 5 wellbeing measures: relaxation, self-awareness, self-confidence, connection with others and positivity about the day ahead. The secondary aims were for doctors to gain personal experience of the psychotherapeutic nature of AT and an understanding of the role of AT in treating mental illnesses.

**Methods:** The workshops were delivered by Art Therapists to 3 different cohorts between October and November 2024. The workshops comprised:

A 10-minute arts-based cross bilateral stimulation exercise.

A 40-minute art making session.

Collective reflection on individual art pieces with psychotherapeutic facilitation.

The pre- and post-workshop surveys included 6 Likert scales measuring wellbeing and understanding of AT, and 2 items exploring perceived personal and professional benefit of AT to doctors. Since the surveys were not labelled for matching, independent samples t-tests (α=0.05) and descriptive statistics were used for analysis.

**Results:** 62 participants completed the pre-workshop survey (55 Resident Doctors and 7 others (Consultants, non-training grade doctors, PAs)); 43 participants completed the post-workshop survey (39 Resident Doctors). All 6 Likert items scores increased post-workshop, with the following being statistically significant:

Relaxation (p <0.001; MD 1.12, 95% CI 0.68 to 1.56);

Self-confidence (p<0.001; MD 0.73, 95% CI 0.31 to 1.15);

Connection with others (p<0.001; MD 0.66, 95% CI 0.30 to 1.01);

Positivity about day ahead (p=0.04; MD 0.46, 95% CI 0.02 to 0.90)

Understanding of art therapy (p<0.001; MD 1.35, CI 1.02 to 1.70).

The increase in self-awareness scores was borderline statistically significant (p=0.06; MD 0.65, 95% CI 0.19 to 1.11).

Post-workshop, there was an increase in the proportion of respondents who felt AT could be beneficial to doctors personally (74% to 98%) and professionally (61% to 93%).

**Conclusion:** Engaging in Art Therapy during work hours has a significantly positive impact on doctors’ wellbeing, with particularly strong evidence for increased relaxation, self-confidence and connection with others. With an overwhelming majority of respondents agreeing it could provide personal and professional benefit to doctors, Psychiatry training schemes would do well to consider arts-based psychotherapeutic approaches within their localities to enhance trainee wellbeing.

